# XiangshaLiujunzi decoction alleviates the symptoms of functional dyspepsia by regulating brain–gut axis and production of neuropeptides

**DOI:** 10.1186/s12906-015-0913-z

**Published:** 2015-10-27

**Authors:** Jing Liu, Feng Li, Xu-Dong Tang, Jie Ma, Xin Ma, Dong-Yu Ge, Gen-Mao Li, Yong Wang

**Affiliations:** School of Preclinical Medicine, Beijing University of Chinese Medicine, Beijing, China; Digestive Department, Xiyuan Hospital, Affiliated with Chinese Academy of TCM, Beijing, China

**Keywords:** Xiangshaliujunzi decoction, Functional dyspepsia, Brain–gut axis, Brain-gut peptide

## Abstract

**Background:**

Chinese medicine xiangshaliujunzi decoction (XSLJZD) plays a key role in treating functional dyspepsia (FD), a common clinical gastrointestinal disorder. However, the mechanism of this disease is unclear. Brain–gut axis regulates food intake behaviour, and this regulatory mechanism is mediated by neuropeptides. Brain–gut axis impairment and neuropeptide alteration may be the pathological mechanisms of FD, and brain–gut axis regulation may influence the action of medicine.

**Methods:**

In our experiment, the effect of XSLJZD on FD was evaluated in terms of food intake, sucrose preference test and electromyogram. Changes in neuropeptides [ghrelin, cholecystokinin (CCK) and vasoactive intestinal polypeptide (VIP)] were detected through immunohistochemistry, real-time PCR and ELISA.

**Results:**

XSLJZD increased food intake and the percentage of sucrose preference (>75 %). However, the response to gastric detention decreased. Furthermore, XSLJZD increased ghrelin, CCK, VIP proteins and genes in the stomach. XSLJZD also increased ghrelin, CCK and VIP proteins in serum. By contrast, XSLJZD decreased the mRNA expression of these neuropeptides in the hypothalamus.

**Conclusions:**

XSLJZD alleviated the symptoms of FD by upregulating the production of ghrelin, CCK and VIP and by increasing the levels of these neuropeptides in circulation. This finding can help elucidate the mechanism of FD and can provide further insight into the pharmacokinetics of XSLJZD.

## Background

Functional dyspepsia (FD) is a common clinical gastrointestinal disorder characterised by persistent or recurrent pain and discomfort. This discomfort is mainly experienced in the upper abdomen without evidence of organic structural abnormalities associated with these symptoms. In a household survey, approximately 25 % of the normal population in the United States suffers from FD [1]. In China, a definite statistics for FD is lacking; however, several works indicate that 8.66 to 11 % of patients opt for hospital admission because of abdominal fullness [2–4]. A number of pathophysiological mechanisms have been proposed to explain several clinical symptoms, including hypersensitivity to gastric distention, impaired gastric accommodation of a meal, delayed gastric emptying, altered duodenal sensitivity to lipids or acid, abnormal duodenojejunal motility and central nervous system (CNS) dysfunction; however, the evidence related to these symptoms varies in patients [5, 6]. Hence, the definite mechanism remains unknown.

FD, an example of functional gastrointestinal disorders, is a common pathological condition affecting the gut, which is controlled by the nervous system [7]. The gastrointestinal tract (GIT) and the nervous system, including CNS and enteric nervous system (ENS), are involved in a two-way extrinsic communication by parasympathetic and sympathetic nerves. These nerves contain efferent fibres and afferent sensory fibres required for gut–brain signalling. Afferent nerves comprise many sensors at the terminals in the gut related to visceral mechano-, chemo-, and noci-receptors; when excited, these sensors may trigger various visceral reflexes that regulate GIT functions, including appetite [8–10]. The disorders affecting the regulation of the two-way communication between the brain and the gut (brain–gut axis) are important in the pathogenesis of these diseases [11]. Neuropeptides are important mediators in the nervous system and between neurons and other cell types. Neuropeptides, such as ghrelin, cholecystokinin (CCK) and vasoactive intestinal polypeptide (VIP), are possibly implicated in the bidirectional gut–brain communication [12]. Ghrelin is a 28-amino acid peptide hormone [13], which is produced predominantly by P/D1 cells of the gastric oxyntic gland; this hormone is mainly found in the proximal stomach [14, 15]. Ghrelin is involved in many biological activities because this hormone plays autocrine and paracrine roles in regulatory processes, such as regulation of appetite, gut motility, growth hormone release, immunomodulation [16, 17] and initiation of food intake under neural control [18]. CCK belongs to the gut–brain family of peptide hormones [19]. This hormone is secreted by the gastrointestinal system in response to food intake; furthermore, CCK is released by specialised neurons in the myenteric plexus and the brain [20]. In a previous study, intravenous injection of CCK suppresses hunger and feeding in humans [21–23]. CCK also participates in signal transduction in the brain–gut axis via the primary afferent fibres of the vagus nerve, and the same fibres probably trigger the expression of receptors for ghrelin [24]. VIP, a 28-amino acid neuropeptide, is distributed in central and peripheral neurons; this neuropeptide is involved in many physiological intestinal functions, such as motility regulation, secretory activity and vasodilatation, peristaltic reflex inhibition in the circular smooth muscle layer and sphincter relaxation [25]. Neuronal VIP is also a mediator of neural response to aspirin-induced stomach inflammatory state [26]. These studies demonstrated that the disorder of the brain–gut axis may be the pathogenesis of FD.

Xiangshaliujunzi decoction (XSLJZD), a classic decoction used during Qing Dynasty in China, plays a key role in the treatment of FD; XSLJZD is more effective than prokinetic drugs in the treatment of this disease [27]. However, the mechanism by which XSLJZD relieves FD remains unknown. We studied the mechanism of the modified XSLJZD on the perspective of the brain–gut axis and neuropeptides. This regulatory mechanism can be the specific mode to treat FD.

## Methods

### Animals

Male Sprague–Dawley rats were used in all of the experiments (SPF Laboratory Animal Technology Co., Ltd., Beijing, China). The experiments were performed in accordance with the Guide for the Care and Use of Laboratory Animals published by the National Institutes of Health (NIH Publications No. 85–23, revised 1996) and with the approval of the Animal Care Committee of Beijing Medical Centre. The 10-day-old rat pups received 0.2 mL of 0.1 % iodoacetamide (IA) in 2 % sucrose by oral gavage daily for 6 days. The control group received 0.2 mL of 2 % sucrose [28]. The 6-week-old IA-treated rats were randomly divided into four groups: model group (*n* = 12; received same volume of water as vehicle), XSLJZD-treated group (*n* = 12; treated with XSLJZD), low-dose XSLJZD-treated group (*n* = 12; treated with half dose of XSLJZD) and domperidone-treated group (*n* = 12). The 6-week-old sucrose-treated rats were designated as the control group (*n* = 12). The 6-week-old rats received 5 mL/kg of each drug or water daily by oral gavage for 10 days.

### Drugs

XSLJZD is composed of eight different Chinese medicinal herbs (Table 1). The components were prepared by the Pharmaceutical Department of Xiyuan Hospital, affiliated with Chinese Academy of TCM. Pure extracts of the components were prepared. The components were dissolved in water. Half dose (12.5 mg/kg) and full dose (25 mg/kg) of XSLJZD were administered to the rats in the low-dose and XSLJZD-treated groups, respectively. Domperidone (3 mg/kg Xian Janssen Pharmaceutical Ltd.) was administered to the rats in the domperidone-treated group. The full dosage of XSLJZD administered to rats was converted from the dosage administered to humans. Meanwhile, the model and control groups received 5 mL/kg water daily by oral gavage for 10 days.

**Table 1:** Components of the XSLJZD solution

Scientific name	Part used	Proportion of ingredients (100 %)
Astragalus mongholicus	Root	12
Codonopsis pilosula	Root	12
Rhizoma Atractylodis Macrocephalae	Rhizome	12
Poria cocos	Sclerotium	12
Fructus Aurantii	Fruit	12
Amomum villosum	Fruit	6.4
Ligusticum chuanxiong Hort.	Sclerotium	9.6
Rhizoma corydalis	Rhizoma	9.6
Medicated Leaven	Fermentation products	12
Glycyrrhiza uralensis Fisch.	Root	2.4

### Food intake measurement

Food intake was measured before and after the drug treatment. After 18 h of fasting, the rats were housed individually. The food was provided for 7 h, and the food consumption was calculated.

### Sucrose preference test (SPT)

SPT [29–31] was conducted before and after the drugs were administered to the rats. Before the test was performed, the rats were treated to adapt to sucrose solution. In the training session, the rats were housed individually for 48 h in a cage with two bottles; one bottle contained 1 % sucrose solution, whereas the other bottle contained tap water. The bottles were placed to the left side and to the right side of the feeding compartment; the positions of these bottles were switched at an interval of 12 h to prevent possible effects of side preference in drinking behaviour. After the training session was completed, only tap water was provided for 6 h. Food and water were then withheld from the rats for 18 h. In the test session, the rats were provided access to two bottles containing 1 % sucrose solution and water for 1 h. Sucrose preference (SP) was quantified with the following equation: SP = [sucrose intake (g)]/[sucrose intake (g) + water intake (g)]. The proportion of rats in each group with an SP value of >75 % was counted and compared through chi-square test.

### Gastric balloon distensions for electromyographic (EMG) testing [28]

After being subjected to overnight fasting, the rats were anesthetised intraperitoneally with 1 % pentobarbital sodium (3 mg/kg) after the drugs were administered for 10 days. Balloons (2.5 cm in length) made from latex condoms were attached to a long catheter. An epigastric incision was made, and the balloon was placed in the stomach through an incision at the tip of the fundus. The pylorus was not obstructed, and no blockage of gastric emptying was observed. A polyethylene tubing to inflate the gastric balloon with air was exteriorised at the back of the neck. Electromyographic (EMG) studies were conducted a week after surgery. Before the experiment, all the animals were anesthetised intraperitoneally with 1 % pentobarbital sodium (3 mg/kg). Then, a pair of stainless steel wires was implanted into the acromiotrapezius (a superficial neck muscle) and externalised at the back of the neck for EMG recordings.

In the experiment, the rats received a series of 20 s gastric balloon distensions: 10, 20, 30, 40 and 50 mmHg (measured with a sphygmomanometer) at an interval of 2 min between distensions. A BL-420S biological and functional experimental system was used to record EMG continuously and to visualise data. EMG was corrected, and the area under the curve was calculated for 20 s distension period. The baseline activity, obtained 20 s before distension, was subtracted from the EMG induced by distension. The data were presented as the change from the baseline as a function of distension pressure.

### Immunohistochemistry

After 10 days of drug treatment, the rats were anesthetised with 1 % pentobarbital sodium. Brains and stomachs were removed, fixed with 10 % formalin and embedded in paraffin. The tissues were subsequently cut in 10 μm sections, mounted on Superfrost Plus slides (1 section/slide) and stored at room temperature. Before the experiment was conducted, the slides were deparaffinised using xylene, subjected to microwave heat-mediated antigen retrieval using citrate buffer at pH 6 for 45 min and were cooled to room temperature. The specimens were immersed in 3 % H_2_O_2_ for 20 min to inactivate endogenous peroxidase and were subsequently washed with PBS (thrice for 2 min each). The sections were incubated overnight at 4 °C with the following antibodies: polyclonal rabbit anti-ghrelin (1:100, Abbiotec), polyclonal rabbit anti-VIP (1:20, Abcam) and polyclonal rabbit anti-CCK-8 (1:100, Abbiotec) in buffer containing 0.01 M PBS. By 9:00 a.m. the next day, the slides were washed with PBS (thrice for 2 min each) and were incubated in Polink-2 Plus® Polymer HRP Detection System (ZSGB-BIO, Beijing) according to the manufacturer’s instructions. Afterwards, the sections were washed thrice with PBS, visualised by using DAB for 10 min at 37 °C and rinsed with running water for 2 min. After dehydration, the slides were mounted using neutral balsam. The sections were visualised under a microscope, and images were acquired using a camera. At least three sections per rat and three rats per group were analysed. Mean integrated optical density (MOD) was calculated using Image-Pro Plus version 6.0 analysis software.

### Real-time PCR

After 10 days of drug treatment, the rats were anesthetised with 10 % chloral hydrate. The hypothalamus and stomachs of these rats were removed. mRNA was quantified by using quantitative RT-PCR. Total RNA was extracted from the rats’ hypothalamus and stomach by using Promega SV total RNA isolation system (Promega, USA) according to the manufacturer’s instructions. Total RNA (2 μg) was reverse-transcribed by using PromegaGoScript (Promega, USA) according to the manufacturer’s instructions. The thermocycling conditions used are listed as follows: initial activation at 95 °C for 30 s; 40 cycles of denaturation at 95 °C for 5 s, annealing at 60 °C for 30 s; and melt curve determination at 65 °C to 95 °C for 5 s. Primers were designed using Primer-BLAST (NCBI, USA) according to the mRNA sequences (by GenBank) of ghrelin (NM_021669.2), VIP (NM_053991.1), CCK (NM_012829.2) and glyceraldehyde-3-phosphate dehydrogenase (GAPDH; NM_017008.4, as control). The PCR products were run on 0.8 % agarose gel to confirm that these products were of the expected size. Results were normalised against GAPDH expression. The primer sequences are listed as follows. The forward and reverse primers of the GAPDH gene were 5′-GGCACAGTCAAGGCTGAGAATG-3′ and 5′-ATGGTGGTGAAGACGCCAGTA-3′, respectively. The forward and reverse primers of the ghrelin gene were 5′-CCAAGGCCATGGTGTCTTCA-3′ and 5′-CTGCAGTTTAGCTGGTGGCTTC-3′, respectively. The forward and reverse primers of the VIP gene were 5′-TCAGTTCCTGGCGATCCTGAC-3′ and 5′-CTCCGCTAAGGCATTCTGCAA-3′, respectively. The forward and reverse primers of the CCK gene were 5′-CCCGATACATCCAGCAGGTC-3′ and 5′-AAATCCATCCAGCCCATGTAGTC-3′, respectively. 2^-ΔΔCt^ was calculated, and differences between groups were analysed by using non-parametric tests.

### ELISA

Rats were anesthetised with 10 % chloral hydrate after 10 days of drug treatment. Blood samples were collected in blank sterile tubes and allowed to clot for 2 h at room temperature. Subsequently, these samples were centrifuged at 1000 rpm for 15 min. Serum was removed and stored at −80 °C. Ghrelin, VIP, and CCK were quantified using specific ELISA kits supplied by CUSABIO (Wuhan, China). Each serum (100 μL) was mixed with sample diluent according to the manufacturer’s instructions. Absorbance was determined at 450 nm.

### Data analysis

All values, except those obtained by sucrose preference test, were presented as means ± SE. One-way ANOVA or non-parametric test was conducted for comparison. Post hoc comparisons were performed using Student–Newman–Keuls test or Mann–Whitney *U* test. Statistical analysis was performed in SPSS 17.0. *P* <0.05 was considered statistically significant.

## Results

### XSLJZD increased the food intake of the rats with FD

**Fig. 1:**
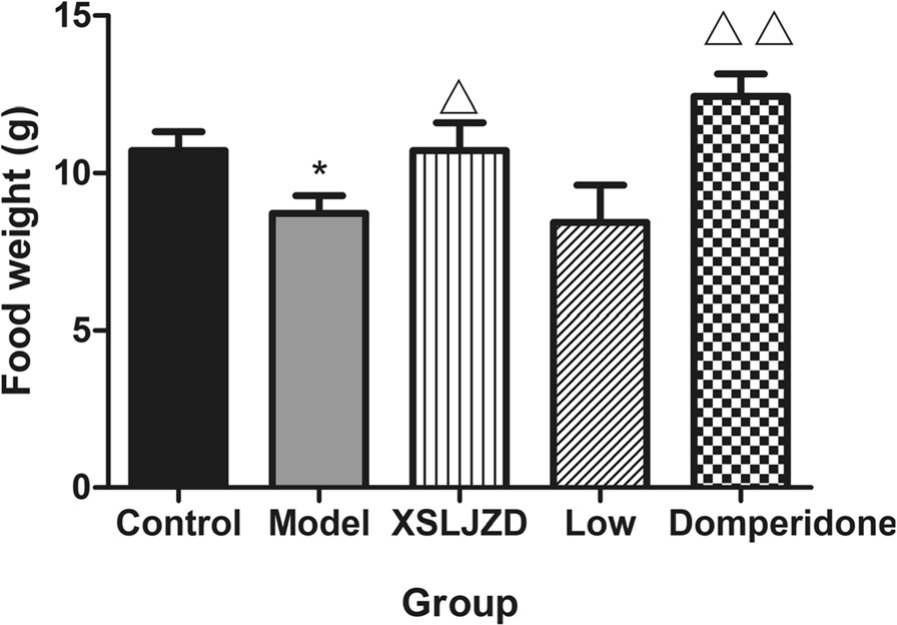
Food intake of each group. Food intake was lower in model group compared with control group. In XSLJZD-treated group and Domperidone-treated group, it was higher compared with model group. * *P* <0.05 compared with the control group; ** *P* <0.01 compared with the control group; △ *P* <0.05 compared with the model group; △△ *P* <0.01 compared with the model group. Data were presented as mean ± SE

After overnight fasting, rats with FD consumed lesser amount of food than the control rats during a 7 h period (10.7 ± 0.6 vs. 8.7 ± 0.5; *P* = 0.01; *n* = 10 in each group). The XSLJZD-treated group consumed greater amount of food than the model group (10.7 ± 0.9 vs. 8.7 ± 0.5; *P* = 0.04; *n* = 10 in each group). Likewise, thedomperidone-treated group consumed greater amount of food than the model group (12.4 ± 0.7 vs. 8.7 ± 0.5; *P* = 0.00). No significant difference was observed between the low-dose XSLJZD-treated group and the model group (8.4 ± 1.2 vs. 8.7 ± 0.5; *P* > 0.1) (Fig. 1).

### XSLJZD increased the percentage of sucrose consumption (>75 %) of rats with FD

**Fig. 2:**
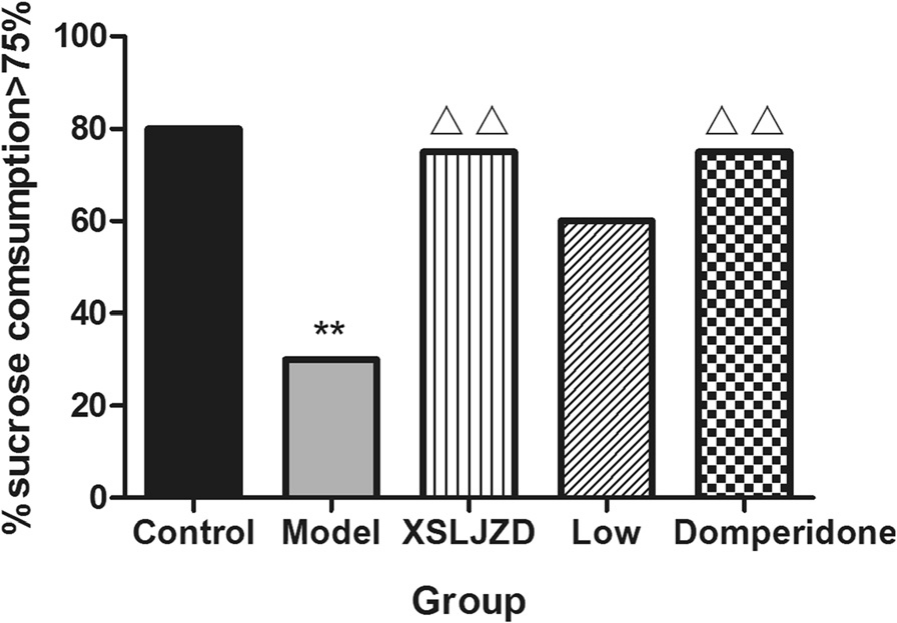
Percentage of rats in each group with sucrose preference (SP) value >75 %. The percentage of SP value >75 % in model group was lower compared with control group. In XSLJZD-treated group and Domperidone-treated group, the percentage was higher compared with model group. ** *P* <0.01 compared with control group and △△ *P* <0.01 compared with model group by Chi-square test. Data were presented as percentage of rats in each group with SP value of >75 %

In the sucrose preference test, no significant difference was observed in terms of sucrose and water intake among the groups (*P* > 0.05). However, the percentage of rats with SP value of >75 % was significantly reduced in rats with FD (30 %) compared with the control rats (80 %; *P* = 0.001; *n* = 10 in each group), as indicated by chi-square test. The percentage significantly increased in the XSLJZD-treated group (75 %) and in the domperidone-treated group (75 %) compared with the rats with FD (30 %; *P* = 0.004; *n* = 10 in each group). The low-dose XSLJZD-treated group did not exhibit significant difference from the model group (Fig. 2).

### XSLJZD reduced the hypersensitivity to gastric distention of rats with FD

**Fig. 3:**
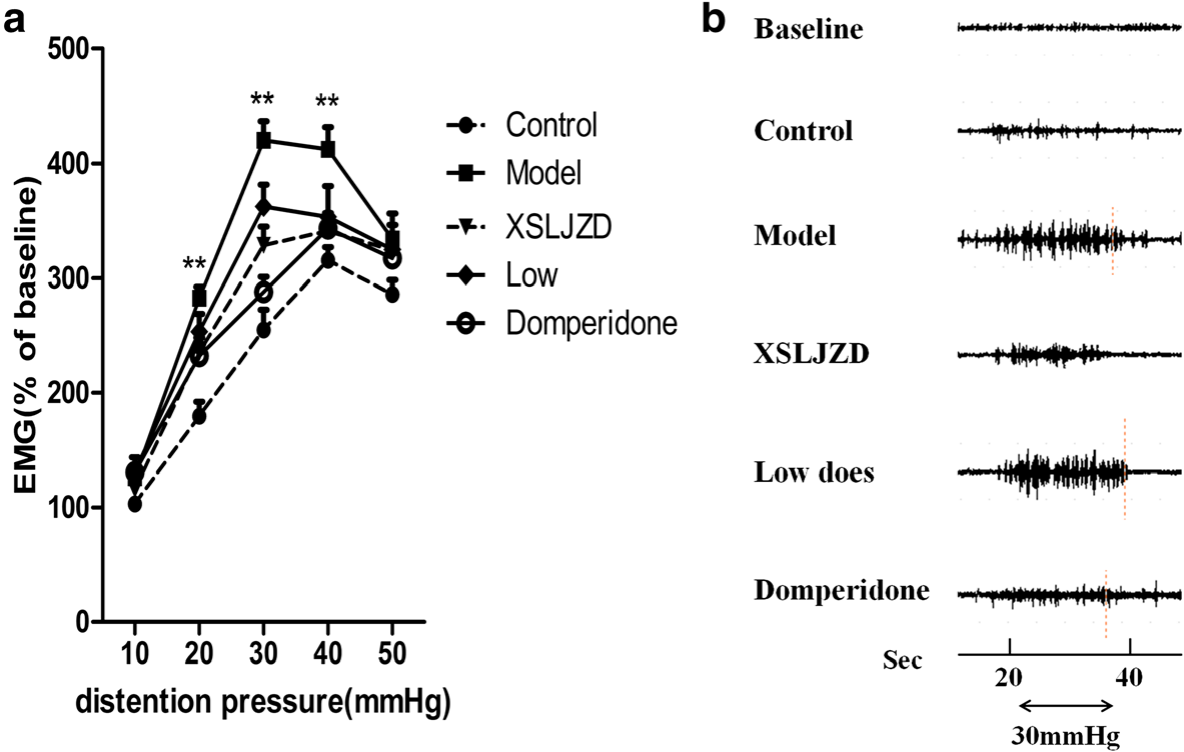
Electromyographic (EMG) response to gastric distention of rats in each group. **a** EMG response to gastric distention of each group at distension of 10 mmHg to 50 mmHg. In XSLJZD-treated group, EMG was reduced significantly compared with model group at distension of 20 mmHg, 30 mmHg and 40 mmHg. ** *P* <0.01 compared with control group. Data were presented as mean ± SE. **b** Representative EMG response at 30 mmHg of each group

After the 10-day treatment, EMS testing was performed. Compared with the control rats, rats with FD significantly increased in EMG at distension pressures of 20 mmHg (179.3 % vs. 282.5 %; *P* = 0.000; *n* = 3 in each group), 30 mmHg (254.9 % vs. 420.1 %; *P* = 0.000) and 40 mmHg (315.4 % vs. 412.3 %; *P* = 0.002). However, no significant difference was observed at 50 mmHg. XSLJZD inhibited the EMG activity of rats with FD to gastric distension in a dose-dependent manner. XSLJZD elicited a significant effect at 20 (277.2 % vs. 282.5 %; *P* = 0.016), 30 (398.3 % vs. 420.1 %; *P* = 0.003) and 40 mmHg (405.5 % vs. 412.3 %; *P* = 0.015). Low dose significantly affected EMG responses only at 30 (362.4 % vs. 420.1 %; *P* = 0.034) and 40 mmHg (353.3 % vs. 412.3 %; *P* = 0.038) compared with the rats with FD, as indicated by one-way ANOVA. Furthermore, the control group did not significantly differ from the domperidone-treatment group (*P* >0.1) (Fig. 3).

### Expression of relative neuropeptides in the brain and the stomach of each group

**Fig. 4:**
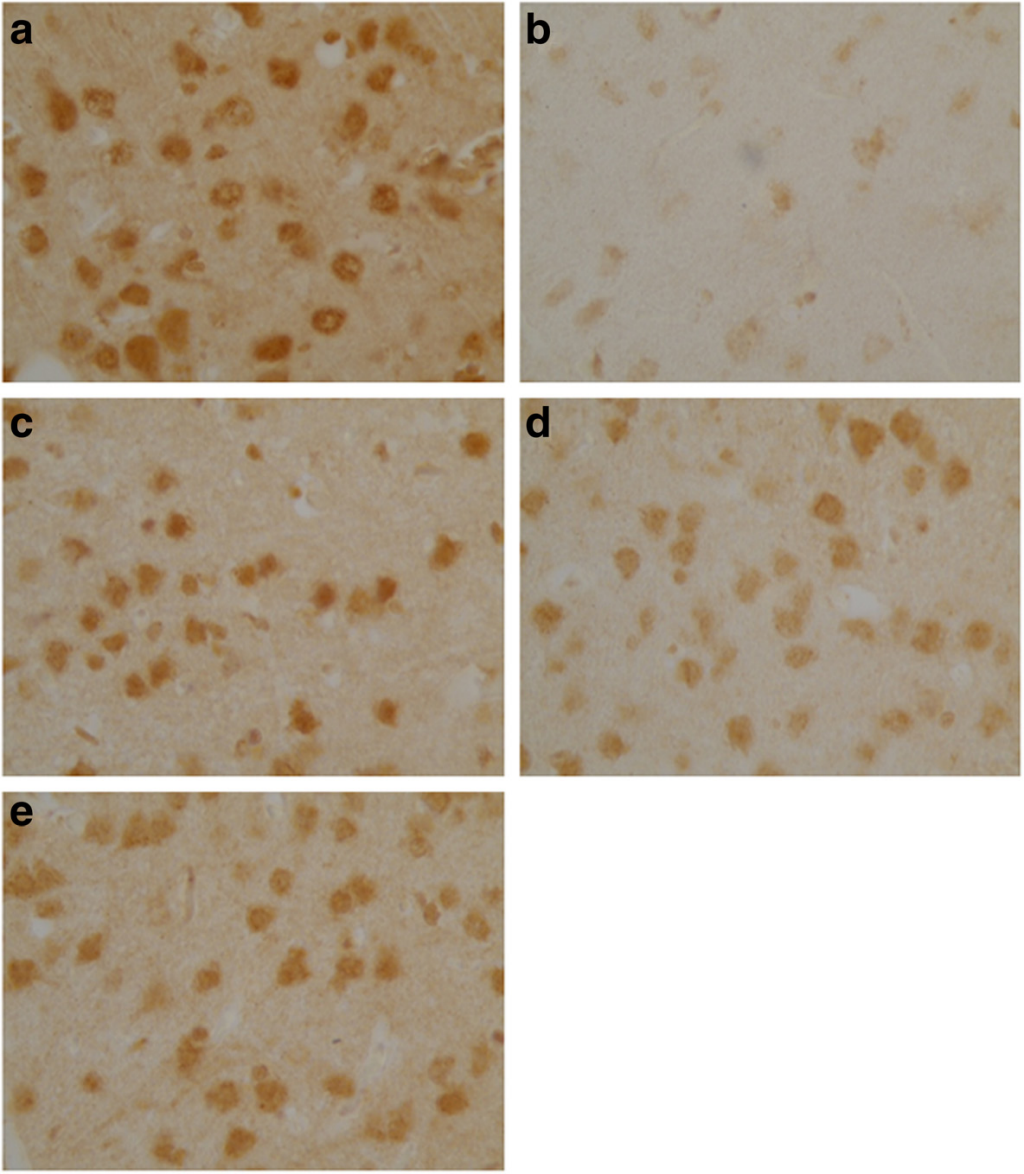
Expression of Ghrelin in hypothalamus of each group. **a** Control group. **b** Model group. **c** XSLJZD-treated group. **d** Low-dose XSLJZD treated group. **e** Domperidone-treated group. Ghrelin distributed in cytoplasm of hypothalamus. (Tissue sections were viewed at 100× magnification.) Positive cells for Ghrelin were brown and circular or pear-shaped. Fewer positive cells can be seen in the model group

**Fig. 5:**
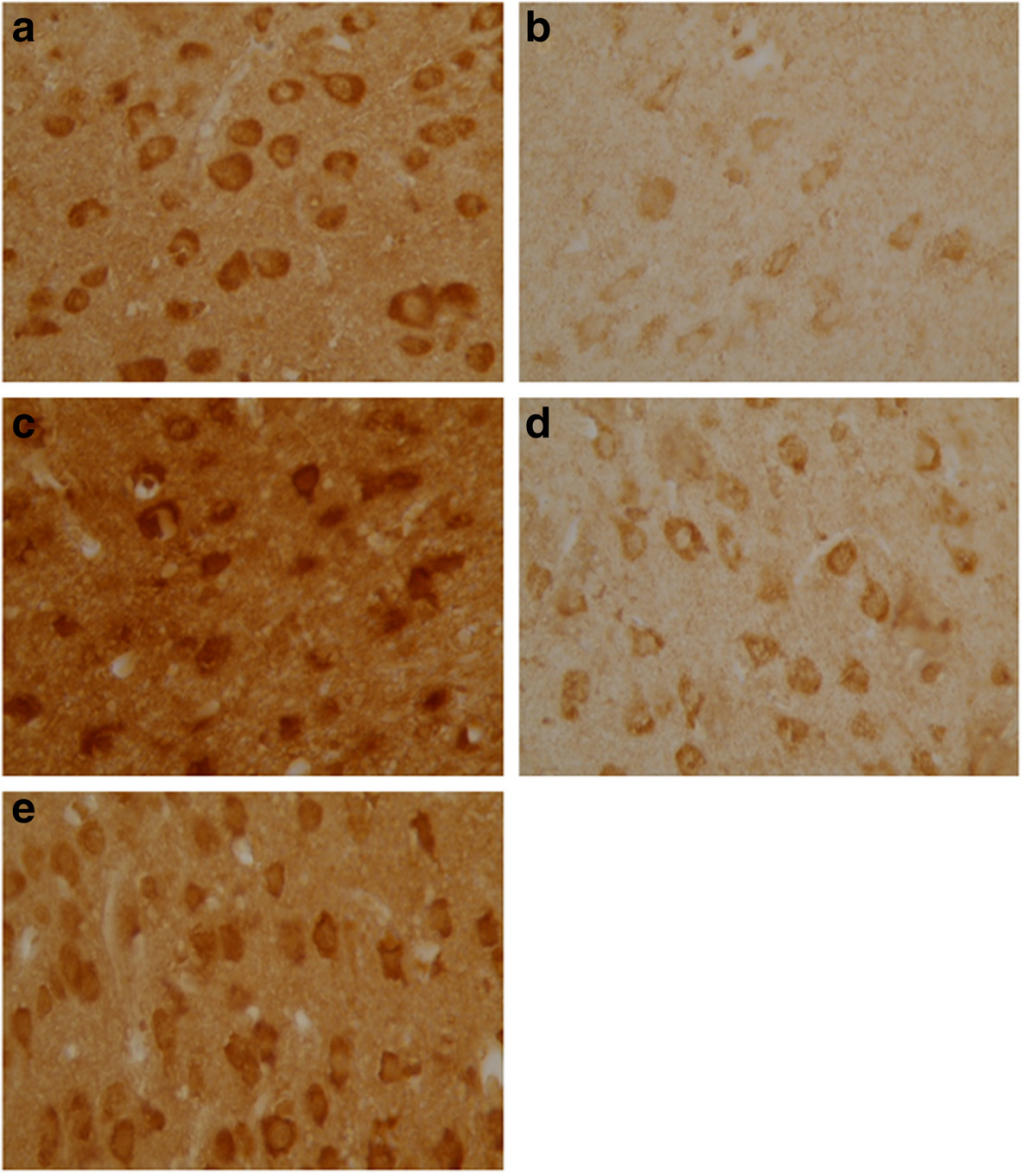
Expression of CCK-8 in hypothalamus of each group. **a** Control group. **b** Model group. **c** XSLJZD-treated group. **d** Low-dose XSLJZD treated group. **e** Domperidone-treated group. CCK-8 mainly distributes in cytoplasm of hypothalamus. (Tissue sections were viewed at 100× magnification.) Positive cells for CCK-8 were brown and circular or oval-shaped. Fewer positive cells can be seen in the model group and low-dose XSLJZD-treated group

**Fig. 6:**
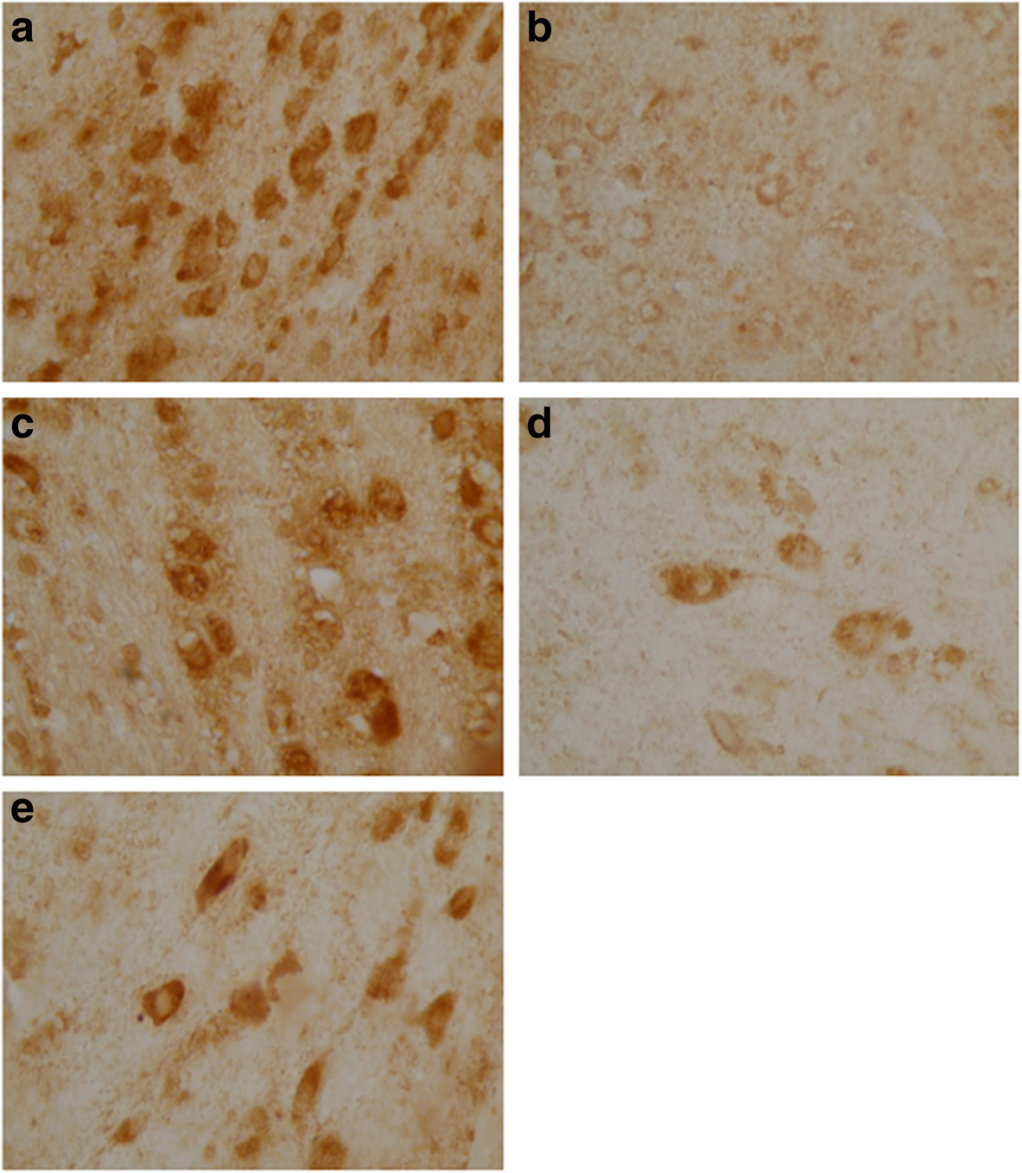
Expression of VIP in hypothalamus of each group. **a** Control group. **b** Model group. **c** XSLJZD-treated group. **d** Low-dose XSLJZD treated group. **e** Domperidone-treated group. Neuropepide VIP mainly distributes in cytoplasm of hypothalamus. (Tissue sections were viewed at 100× magnification.) Positive cells for VIP were brown and circular or oval-shaped. Fewer positive cells can be seen in the model group and low-dose XSLJZD-treated group and domperidone-treated group

**Fig. 7:**
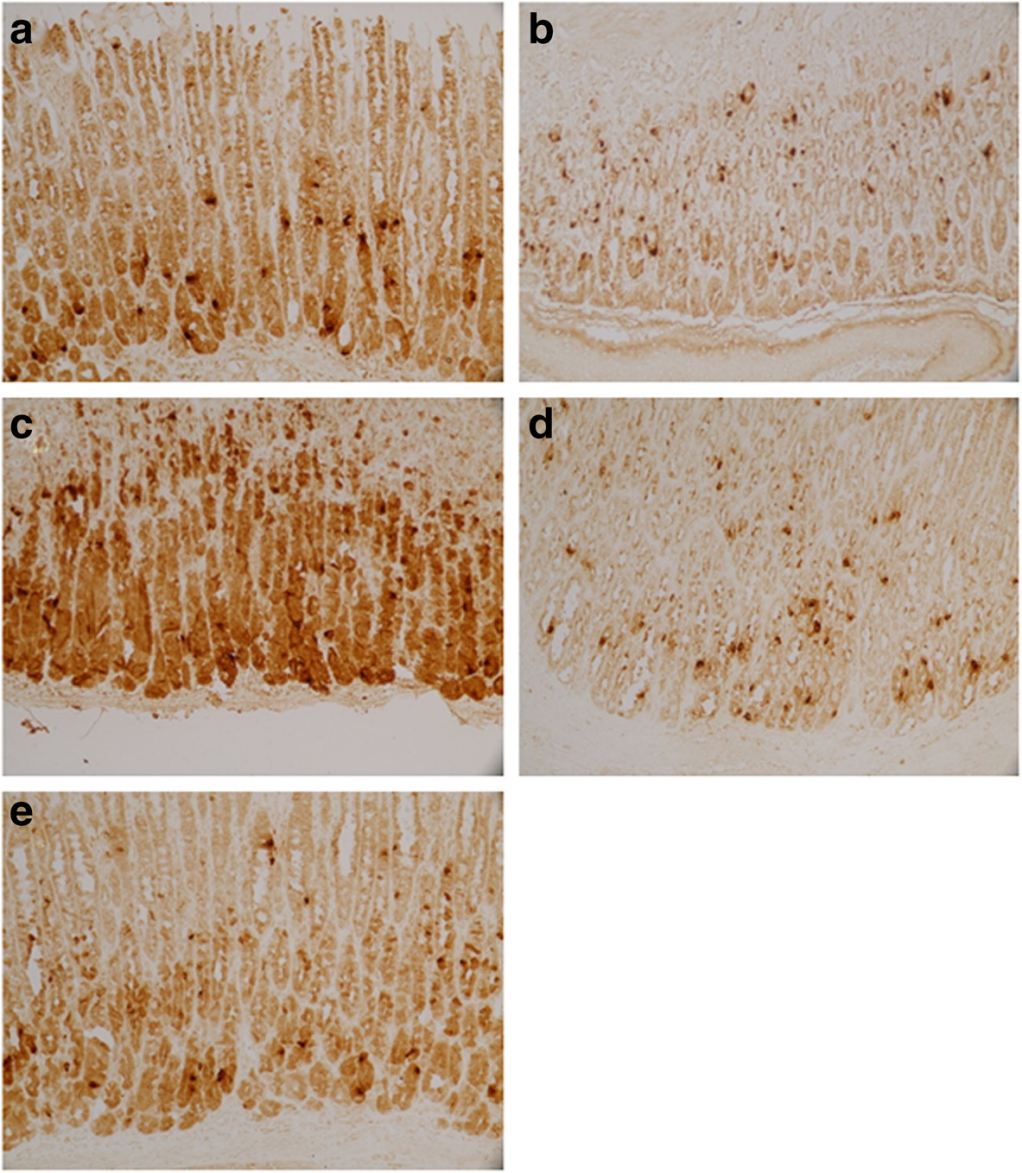
Expression of Ghrelin in stomach of each group. **a** Control group. **b** Model group. **c** XSLJZD-treated group. **d** Low-dose XSLJZD treated group. **e** Domperidone-treated group. Ghrelin was expressed as granular neuropetide in the cytoplasm of the stratum basale of the stomach (Tissue sections were viewed at 20× magnification.) Brown cells were positive for Ghrelin. Model group expressed lower Ghrelin than control group, XSLJZD significantly increased ghrelin of rats with FD

**Fig. 8:**
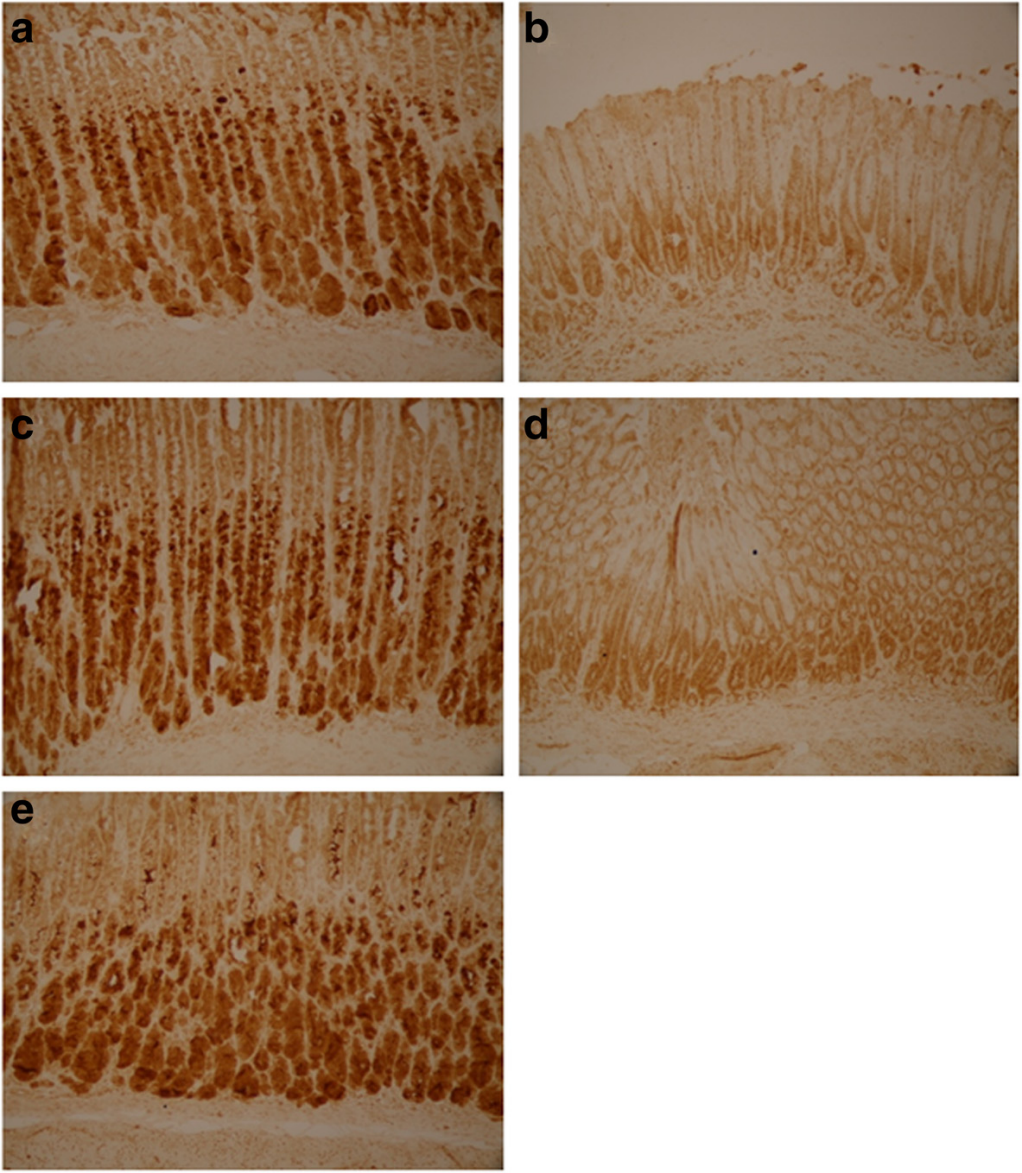
Expression of CCK-8 in stomach of each group. **a** Control group. **b** Model group. **c** XSLJZD-treated group. **d** Low-dose XSLJZD treated group. **e** Domperidone-treated group. CCK-8 mainly distributes in stratum basale of stomach. (Tissue sections were viewed at 20× magnification.) Positive CCK-8 expressed as brown granule, distributed in cytoplasm. Expression of CCK-8 was lower in model group and low-dose XSLJZD-treated group, higher in control group, XSLJZD-treated group and domperidone-treated group

**Fig. 9:**
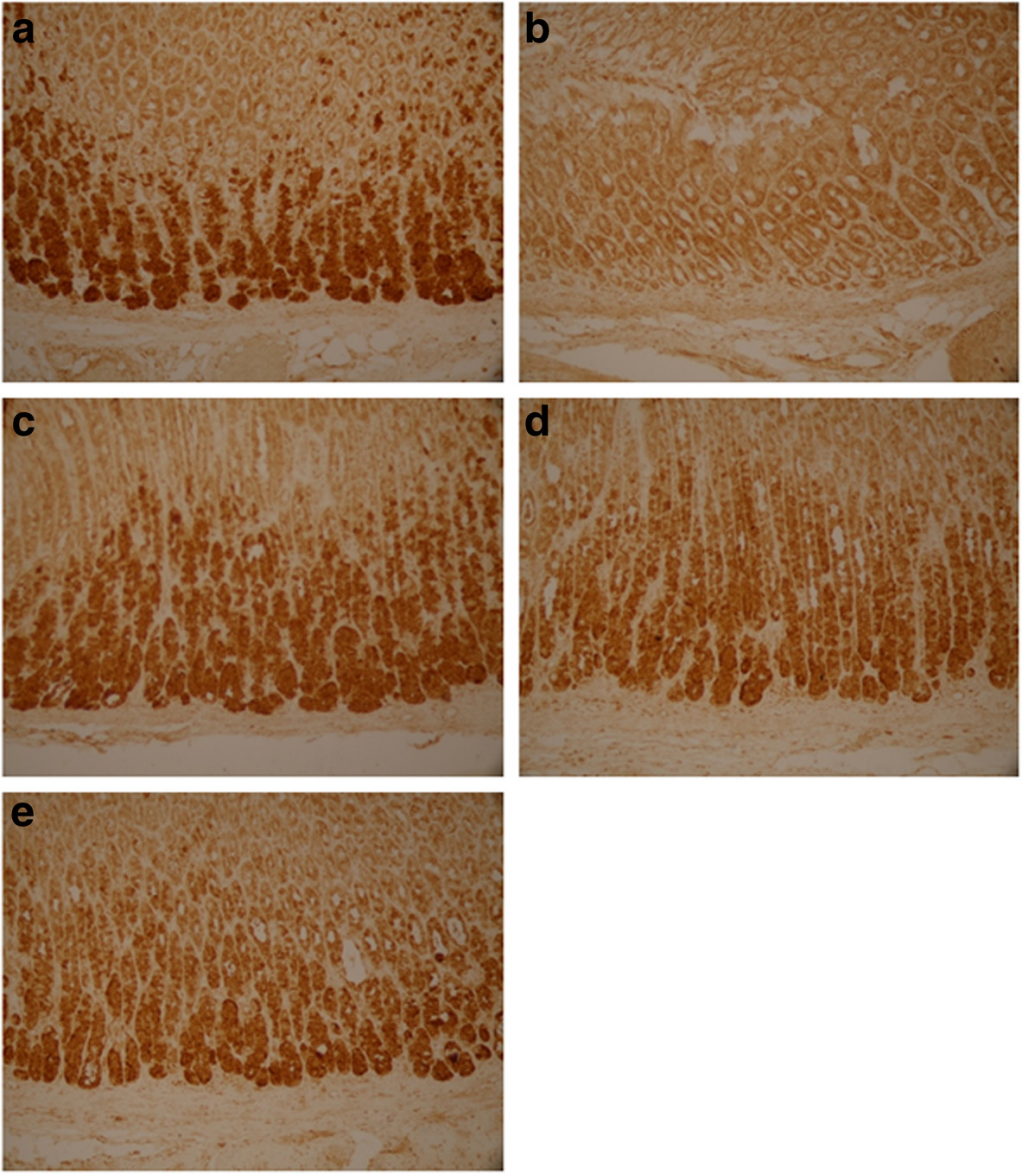
Expression of VIP in stomach of each group. **a** Control group. **b** Model group. **c** XSLJZD-treated group. **d** Low-dose XSLJZD treated group. **e** Domperidone-treated group. VIP was expressed as granular neuropetide in the cytoplasm of the stratum basale of the stomach (Tissue sections were viewed at 20× magnification.) Brown cells were positive for VIP. Expression of VIP was lower in model group and higher in control group, XSLJZD-treated group and domperidone-treated group

**Fig. 10:**
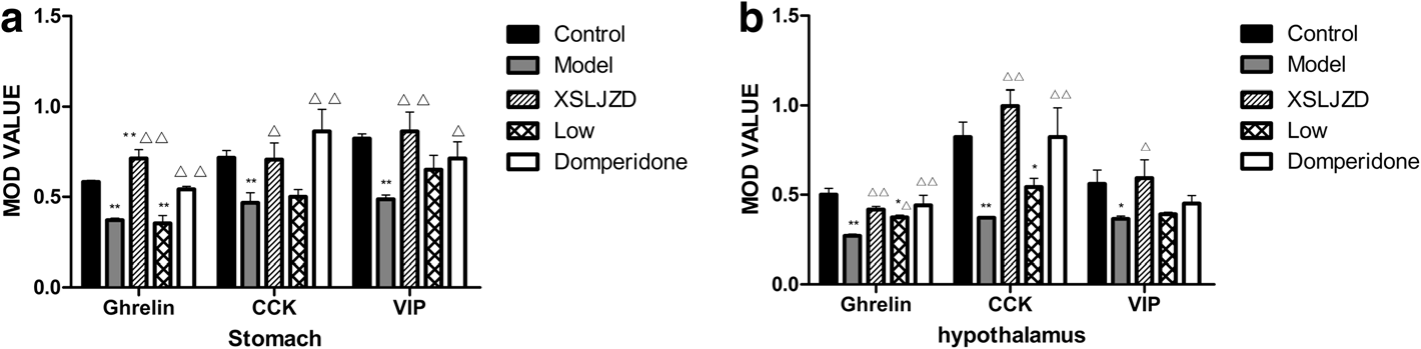
Mean integrated optical density (MOD) value of relative neuropeptides. **a** MOD value of neuropeptides in the stomach. **b** MOD value of neuropeptides in hypothalamus. * *P* <0.05 compared with control group; ** *P* <0.01 compared with control group. △ *P* <0.05 compared with the model group; △△ *P* <0.01 compared with the model group. Data were presented as mean ± SE

Ghrelin, CCK-8 and VIP were expressed as granular neuropeptides in the cytoplasm of the hypothalamus (Figs. 4, 5 and 6) and the stratum basale of the stomach (Figs. 7, 8 and 9). The expressions of these neuropeptides were lower in the brain and in the stomach of rats with FD compared with those of the control rats. In the stomach and the hypothalamus, ghrelin, CCK-8 and VIP of the rats with FD were lower than those of the control rats (*P* <0.05; *n* = 3 in each group). Low-dose XSLJZD-treated groups did not significantly differ from the model group. However, XSLJZD significantly increased ghrelin, CCK-8 and VIP of rats with FD (*P* <0.05; *n* = 3). Ghrelin, CCK-8 and VIP of the domperidone-treated group were also higher than those of the model group, except for VIP in the hypothalamus (*P* <0.05; *n* = 3) (Table 2 and Fig. 10).

**Table 2:** MOD value of relative neuropeptides in the stomach and hypothalamus of each group

Part	Groups	Ghrelin	CCK-8	VIP
Stomach	Control	0.583 ± 0.008	0.714 ± 0.042	0.823 ± 0.025
	Model	0.368 ± 0.010^**^	0.467 ± 0.057^*^	0.486 ± 0.025^**^
	XSLJZD	0.716 ± 0.050^*△△^	0.706 ± 0.090^△^	0.861 ± 0.107^△△^
	Low dose XSLJZD	0.356 ± 0.044^**^	0.498 ± 0.039	0.652 ± 0.082
	Domperidone	0.543 ± 0.163^△△^	0.863 ± 0.122^△△^	0.711 ± 0.092^△^
Hypothalamus	Control	0.497 ± 0.036	0.825 ± 0.081	0.561 ± 0.077
	Model	0.268 ± 0.010^**^	0.372 ± 0.006^**^	0.365 ± 0.017^*^
	XSLJZD	0.417 ± 0.017^△△^	0.995 ± 0.088^△△^	0.594 ± 0.105^△^
	Low dose XSLJZD	0.372 ± 0.011^*△^	0.540 ± 0.050^*^	0.391 ± 0.012
	Domperidone	0.441 ± 0.059^△△^	0.824 ± 0.163^△△^	0.449 ± 0.046

*P <0.05 compared with the control group; **P <0.01 compared with the control group; ^∆^ *P* <0.05 compared with the model group; ^∆∆^ *P* <0.01 compared with the model group

### XSLJZD increased the neuropeptides in the serum of rats with FD

**Fig. 11:**
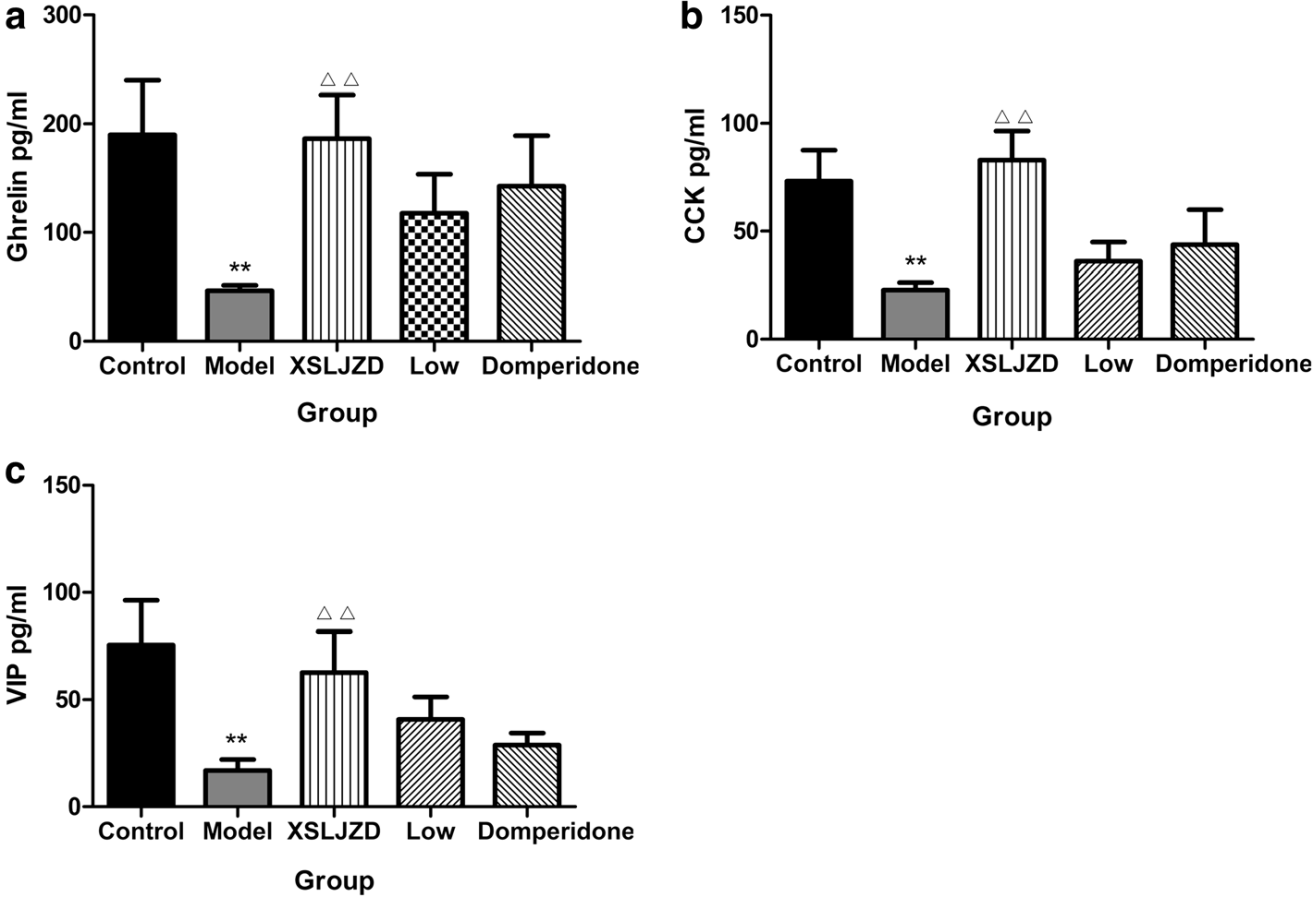
Expression of relative neuropeptides in the serum. **a** Expression of Ghrelin of each group. **b** Expression of CCK of each group. **c** Expression of VIP of each group. ** *P* <0.01 compared with the control group; ∆∆ *P* <0.01 compared with the model group . Data were presented as mean ± SE

Ghrelin was reduced in the serum of rats with FD compared with that of the control rats (46.72 ± 4.92 vs. 189.9 ± 49.96; *P* = 0.007; *n* = 7 in each group). By contrast, ghrelin was increased in the XSLJZD-treated group compared with the model group (186.4 ± 40.13 vs. 46.72 ± 4.92; *P* = 0.009; *n* = 7 in each group). No significant difference was observed among the control group, low-dose XSLJZD-treated group and domperidone-treated group in terms of ghrelin in the serum. Similar to ghrelin, CCK was reduced in the serum of rats with FD compared with that of the control rats (22.77 ± 3.59 vs. 77.19 ± 14.36; *P* = 0.003; *n* = 7 in each group). CCK was increased by XSLJZD in a dose-dependent manner. The low-dose XSLJZD-treated groups did not significantly differ with the rats with FD in terms of CCK in the serum (*P* >0.10); however, the XSLJZD-treated group exhibited a significant increase in serum CCK compared with that of rats with FD (82.97 ± 13.47 vs. 22.77 ± 3.59; *P* = 0.001; *n* = 7 in each group). VIP was reduced in the serum of rats with FD (16.95 ± 5.15 vs. 75.61 ± 20.12; *P* = 0.003; *n* = 7 in each group) compared with that in the control group. VIP in the XSLJZD-treated group significantly increased compared with that in the model group (62.71 ± 19.05 vs. 16.95 ± 5.15; *P* = 0.017; *n* = 7 in each group). No significant difference in VIP of low-dose XSLJZD-treated groups and domperidone-treated groups was observed. Nevertheless, a moderate dose of XSLJZD could increase VIP in the serum (Fig. 11).

### XSLJZD increased the gene expression of the relative neuropeptides in the stomach

**Fig. 12:**
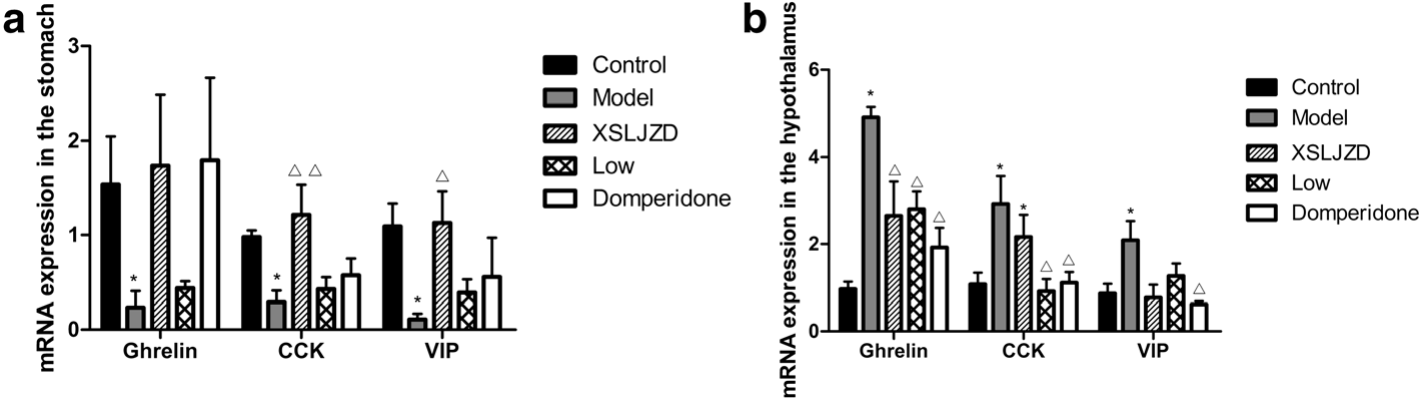
mRNA expression of neuropeptides in the stomach (**a**) and hypothalamus (**b**) of each group. * *P* <0.05 compared with the control group; ** *P* <0.01 compared with the control group; ∆ *P* <0.05 compared with the model group; ∆∆ *P* <0.01 compared with the model group by nonparametric test. Data were presented as mean ± SE

In the stomach, the mRNA expressions of ghrelin, CCK and VIP of rats with FD were lower compared with those of the control rats (*P* <0.05; *n* = 6 in each group). The mRNA expressions of CCK and VIP of the XSLJZD-treated group were higher than those of the model group. No significant difference in ghrelin expression was observed in the model and drug-treated groups. Nevertheless, moderate-dose XSLJZD increased ghrelin mRNA expression in the stomach. In the hypothalamus, the mRNA expressions of ghrelin, CCK and VIP of rats with FD were higher than those of the control rats (*P* <0.05; *n* = 6 in each group). The mRNA expression of ghrelin in the XSLJZD-treated and low-dose XSLJZD-treated groups as well as in the domperidone-treated group was reduced compared with that of the model group (*P* <0.05). CCK mRNA expression in the low-dose XSLJZD-treated group and domperidone-treated group was lower (*P* <0.05) than that of the model group. Furthermore, VIP mRNA expression in thedomperidone-treated group was reduced (*P* <0.05) compared with that of the model group. The mRNA expression of these neuropeptides was not altered in a dose-dependent manner. However, the mRNA expressions of ghrelin, CCK and VIP in the hypothalamus of rats with FD were reduced by XSLJZD (Table 3 and Fig. 12).

**Table 3:** The mRNA expression of relative nouropeptides in the stomach and hypothalamus of each group

Part	Groups	Ghrelin	CCK	VIP
Stomach	Control	1.536 ± 0.509	0.981 ± 0.069	1.092 ± 0.244
	Model	0.232 ± 0.172^*^	0.291 ± 0.121^*^	0.111 ± 0.059^*^
	XSLJZD	1.735 ± 0.747	1.212 ± 0.320^△△^	1.127 ± 0.336^△^
	Low dose XSLJZD	0.439 ± 0.074	0.428 ± 0.125	0.394 ± 0.139
	Domperidone	1.797 ± 0.869	0.637 ± 0.095	0.555 ± 0.417
hypothalamus	Control	0.967 ± 0.172	1.075 ± 0.268	0.871 ± 0.222
	Model	4.917 ± 0.239^*^	2.923 ± 0.640^*^	2.084 ± 0.442^*^
	XSLJZD	2.646 ± 0.797^△^	2.162 ± 0.511^*^	0.777 ± 0.295
	Low dose XSLJZD	2.804 ± 0.407^△^	0.916 ± 0.281^△^	1.268 ± 0.285
	Domperidone	1.925 ± 0.448^△^	1.113 ± 0.247^△^	0.617 ± 0.073^△^

*P <0.05 compared with the control group; ^∆^ *P* <0.05 compared with the model group; ^∆∆^ *P* <0.01 compared with the model group

## Discussion

Our findings demonstrated the mechanism by which the Chinese medicine XSLJZD relieved FD. Previous studies revealed that FD is associated with gastric hypersensitivity to mechanical distention and impaired accommodation [28, 32]. Gastric hypersensitivity or motor change can be caused by impairment, such as gastric inflammation or mechanical impairment, which can occur during childhood; the change remains, but the impairment was resolved completely [33, 34]. Previous studies showed that iodoacetamidecan induce mild damage to the gastric surface and chronically sensitise gastric sensory afferents during adulthood in the absence of overt structural abnormalities. A rat model displayed the symptoms of functional dyspepsia similar to that experienced by human beings [28]. Therefore, iodoacetamide-treated rats as FD model are used to study the effects and mechanisms of Chinese medicine XSLJZD.

Studies provided insights into the biological basis of this form of neonatal programming on the brain–gut axis [10], and they also demonstrated the release of neuropeptides such as ghrelin, CCK and VIP. Ghrelin, CCK and VIP are produced in the stomach and then released into the circulation [14, 20, 25]. The arcuate nucleus (ARC) of the hypothalamus is implicated in food intake regulation and energy homeostasis. ARC is adjacent to the median eminence, which is a ‘circumventricular organ’ with fenestrated capillaries and an incomplete blood–brain barrier [35]. Circulating gut hormones can pass across the median eminence and directly affect the activity of ARC neurons. The hypothalamus receives both hormonal signals and peripheral neurons, and then relay information regarding energy availability [36]. Hormonal signals from the periphery are integrated with high brain centre signals to regulate appetite and control energy expenditure. The release of gut hormones, such as ghrelin, CCK and VIP, regulates the brain–gut axis using hormonal signals [16, 17, 20, 26].

In our study, XSLJZD could increase the food intake of FD rats. A decrease in sucrose solution consumption manifests anhedonia, which is the decreased ability to experience pleasures [30, 37], because of its association with drink and food intake; this decrease also reflects the appetite of rats. In our experiment, model rats showed low percentage of SP value at >75 %; XSLJZD-induced increase in SP percentage corresponded to the improvement of the appetite of rats with FD. Early satiation or postprandial fullness or pain in the upper gastrointestinal origin is the prominent feature observed in FD patients. Those symptoms in animals are conceptually manifested as gastric hypersensitivity to mechanical distention in the absence of overt morphological or histological changes. In FD patients, the presence of visceral hypersensitivity to graded gastric distention is possibly correlated with postprandial pain [38, 39]; other non-painful symptoms, such as nausea, satiety and fullness, are also triggered at lower distension pressures. This result suggests that multimodal pathways are sensitized [40]. We reproduced this hypersensitivity in our experiment [28, 32, 41] and calculated the EMG to graded gastric distention at pressures ranging from 10 mmHg to 50 mmHg. Rats with FD displayed significantly higher EMG in response to gastric distention at 20, 30 and 40 mmHg. Our results also showed that XSLJZD inhibited visceromotor responses caused at distention pressures of 30 and 40 mmHg. Previous studies also demonstrated that neonatal IA-treated rats display significantly higher behavioural scores in response to gastric distension at 40, 60 and 80 mmHg [28]. In another study, rats with FD exhibit a significantly higher EMG in response to gastric distension at 50 mmHg to 120 mmHg with the same result [33]. This difference may be attributed to the instrument used in this study. We observed a dose-dependent effect of XSLJZD on food intake, sucrose preference test and response to mechanical gastric distension. Low dose elicited slight or no effect, and XSLJZD caused a more robust effect. According to the Rome III criteria, epigastric pain or buming, postprandial fullness and early satiation are symptoms of FD [42]. XSLJZD could alleviate the main symptoms of FD in our experiment.

In the experiment, transient gastric irritation in the neonatal period could result in impaired brain–gut axis and decrease some neuropeptides. Result manifested the strong association between FD and the brain–gut axis.

We also examined the effects of transient neonatal gastric inflammation on ghrelin, CCK and VIP expressions; the brain–gut axis response to stress; and the mechanism of XSLJZD on FD. The expressions of ghrelin, CCK and VIP in the stomach and in the serum were significantly lower in IA-treated rats compared with those in control rats. These neuropeptides were significantly increased by XSLJZD. The effect of XSLJZD on the neuropeptides was dose dependent. In particular, XSLJZD was more effective than the low dose. The expression of ghrelin, CCK and VIP were also significantly decreased in the hypothalamus of rats with FD compared with those of the control rats. However, the mRNA expressions of these neuropeptides were increased. XSLJZD increased the protein expression but decreased the mRNA expression of these neuropeptides in the hypothalamus. Ghrelin, CCK and VIP were produced in the stomach and then released into the blood [14, 20, 25]. In our experiment, XSLJZD could increase the production of these neuropeptides, thereby increasing the neuropeptide levels in circulation. The decrease in the mRNA expression of these neuropeptides in the hypothalamus maybe considered as feedback regulation.

## Conclusions

Our results demonstrated that rats with FD with transient gastric irritation in the neonatal period showed brain–gut axis impairment and low neuropeptide level. Chinese medicine XSLJZD could alleviate the symptoms of FD and upregulate the brain–gut axis by increasing the production of neuropeptides such as ghrelin, CCK, and VIP. These findings have major implications in the mechanism of XSLJZD to relieve FD, which would help investigate FD mechanism and pharmacokinetics.

## Abbreviations

XSLJZD: Xiangsha Liujunzi decoction
FD: Functional dyspepsia
CCK: Cholecystokinin
VIP: Vasoactive intestinal polypeptide
GIT: Gastrointestinal tract
CNS: Central nervous system
ENS: Enteric nervous system
SP: Sucrose preference
EMG: Electromyographic
MOD: Mean integrated optical density
ARC: Arcuate nucleus
